# Association of Cabazitaxel Treatment Beyond 10 Cycles with Survival Outcomes in Metastatic Castration-Resistant Prostate Cancer: A Retrospective 8-Month Landmark Analysis

**DOI:** 10.3390/jcm15186966

**Published:** 2026-09-08

**Authors:** Hakan Taban, Sercan Aksoy, Yasemin Evlendi, Burak Yasin Aktaş, Ferit Aslan, Mustafa Erman

**Affiliations:** 1 Medical Oncology Clinic, Medical Park Ankara Hospital, Ankara 06680, Turkey; feritferhat21@gmail.com; 2 Department of Medical Oncology, Hacettepe University Cancer Institute, Ankara 06100, Turkey; saksoy07@yahoo.com (S.A.); dryasemen13@gmail.com (Y.E.); byaktas@hotmail.com (B.Y.A.); 3 Department of Preventive Oncology, Hacettepe University Cancer Institute, Ankara 06100, Turkey; ermanm1968@gmail.com

**Keywords:** cabazitaxel, immortal time bias, landmark analysis, metastatic castration-resistant prostate cancer, overall survival, retrospective study, treatment duration

## Abstract

**Background/Objectives:** Cabazitaxel is an established treatment option for patients with metastatic castration-resistant prostate cancer (mCRPC), but outcomes beyond the conventional 10-cycle threshold remain incompletely characterized. This threshold largely reflects clinical trial design. We therefore evaluated outcomes associated with cabazitaxel treatment beyond 10 cycles using a predefined 8-month landmark analysis intended to reduce early immortal time bias. **Methods:** This retrospective single-center study included 98 patients with mCRPC treated with cabazitaxel between 2010 and 2020. The final cohort comprised 74 patients who were alive at the predefined 8-month landmark. The primary endpoint was overall survival (OS) from the landmark, and secondary endpoints included conditional radiologic progression-free survival (rPFS), conditional prostate-specific antigen progression-free survival (PSA-PFS), treatment response, and safety. **Results:** Among the 74 patients included in the landmark cohort, 45 (60.8%) ultimately received ≤10 cycles and 29 (39.2%) received >10 cycles. The median landmark OS was numerically longer in the >10-cycle group (17.6 vs. 10.4 months; *p* = 0.123). Conditional rPFS and PSA-PFS did not differ significantly between groups (>10 vs. ≤10 cycles: 4.4 vs. 2.3 months; *p* = 0.255 and 4.2 vs. 2.2 months; *p* = 0.137, respectively). In a broader baseline-adjusted sensitivity model, the treatment duration association was not statistically significant (HR for ≤10 vs. >10 cycles, 1.58; 95% CI, 0.91–2.74; *p* = 0.105). The crude cumulative proportions of grade ≥3 neutropenia and febrile neutropenia were higher in the >10-cycle group. **Conclusions:** Cabazitaxel treatment beyond 10 cycles appeared feasible in selected patients. However, future-derived exposure classification and responder enrichment limit causal interpretation. The survival association was not consistently maintained across sensitivity analyses, supporting a hypothesis-generating interpretation rather than evidence of a survival benefit from continuation beyond 10 cycles.

## 1. Introduction

Prostate cancer is the second most frequently diagnosed cancer and a leading cause of cancer-related death among men worldwide [1]. Although most patients present with localized disease, some develop metastatic recurrence and ultimately progress to metastatic castration-resistant prostate cancer (mCRPC), which remains associated with substantial morbidity and poor survival despite major therapeutic advances [2,3]. Current treatment options include androgen receptor pathway inhibitors (ARPIs), taxane-based chemotherapy, poly(ADP-ribose) polymerase inhibitors, prostate-specific membrane antigen-targeted radioligand therapy, and other biomarker-directed approaches [4,5]. Guidelines recommend cabazitaxel as an important option for patients with mCRPC previously exposed to docetaxel and an ARPI [2,4,5,6].

Cabazitaxel is a second-generation taxane developed to overcome resistance associated with prior taxane exposure [7]. The phase III TROPIC trial introduced cabazitaxel into clinical practice by demonstrating improved overall survival (OS) versus mitoxantrone in patients with mCRPC previously treated with docetaxel; treatment was limited to a maximum of 10 cycles [8]. PROSELICA subsequently showed that cabazitaxel 20 mg/m^2^ was non-inferior to 25 mg/m^2^ for OS and had a more favorable safety profile [9], whereas CARD demonstrated improved progression-free survival (PFS) and OS versus an alternative ARPI in patients previously treated with docetaxel and an ARPI [6]. Real-world studies have also supported its effectiveness and manageable safety profile [10,11].

Current international guidelines recommend cabazitaxel for appropriately selected patients with mCRPC but do not specify a fixed maximum number of treatment cycles [2,4,5,12]. A 10-cycle limit is not defined as a mandatory stopping point, and treatment beyond 10 cycles is not specifically recommended as a standard strategy. The conventional 10-cycle threshold largely reflects the design of pivotal trials rather than an evidence-based requirement to discontinue treatment [8,9]. In clinical practice, treatment duration is individualized according to response, tolerability, patient preference, physician judgment, and reimbursement policies [13]. Evidence regarding cabazitaxel treatment beyond 10 cycles in patients with ongoing disease control remains limited [13,14,15]. Small retrospective studies have suggested that treatment beyond 10 cycles is feasible and may be associated with longer PFS and OS in selected patients [13,14,15]. Noronha et al. reported sustained clinical benefit with manageable toxicity [15], while Shiota et al. and Al-Mansouri et al. observed more favorable survival outcomes with treatment beyond 10 cycles [13,14].

These findings require cautious interpretation because treatment duration analyses are susceptible to selection bias and immortal time bias [16]. Patients who receive >10 cycles must survive long enough and remain sufficiently fit to continue treatment, potentially creating an apparent survival advantage. Moreover, previous studies included relatively small cohorts and generally did not apply methods to reduce immortal time bias [13,14,15].

To address this gap, we evaluated clinical outcomes associated with cabazitaxel treatment beyond 10 cycles in patients with mCRPC using a predefined 8-month landmark analysis. This approach was intended to reduce early immortal time bias while recognizing that treatment groups remained defined by the total number of cycles ultimately received. This study therefore aimed to characterize differences between treatment trajectories rather than to establish a causal benefit of treatment continuation beyond 10 cycles.

## 2. Materials and Methods

### 2.1. Study Design and Patient Population

This retrospective single-center study was conducted at Hacettepe University Cancer Institute. Medical records of patients with prostate cancer treated between 1 January 2010, and 31 December 2020, were reviewed. Patients with mCRPC who received at least one cycle of cabazitaxel and had adequate clinical and follow-up data were eligible. Patients with non-adenocarcinoma histology, another active malignancy, or insufficient data were excluded. 

Castration-resistant prostate cancer was defined according to Prostate Cancer Working Group 2 (PCWG2) recommendations as biochemical, radiologic, or clinical progression despite ongoing androgen deprivation therapy and a castrate serum testosterone level (<50 ng/dL) [17].

A predefined 8-month landmark design was applied to reduce early immortal time bias related to treatment duration by restricting the primary analysis to patients who were alive at a common time point after cabazitaxel initiation [16,18]. Of 98 initially identified patients, 74 were alive at the landmark and constituted the final cohort. Patients were classified according to the total number of cabazitaxel cycles ultimately received as ≤10 or >10 cycles, consistent with the conventional threshold and previous retrospective studies [13,14]. Because exposure groups were defined by the total number of cycles ultimately received, including post-landmark treatment in some patients, the analysis was specified as a conditional prognostic comparison rather than a comparison of treatment strategies assigned at the landmark. Exact cycle-level administration dates and treatment interruptions were unavailable; therefore, the 8-month landmark was not considered a precise proxy for a clinical decision point near cycle 10.

This study was conducted in accordance with the Declaration of Helsinki and approved by the Hacettepe University Non-Interventional Clinical Research Ethics Committee (approval no: GO 21/1343; 21 December 2021). Informed consent was waived because of the retrospective design and use of anonymized data. This study was reported in accordance with the Strengthening the Reporting of Observational Studies in Epidemiology (STROBE) guidelines.

### 2.2. Data Collection

Clinical, pathologic, laboratory, and treatment-related data were collected from electronic medical records. Variables included age at prostate cancer diagnosis and at cabazitaxel initiation, Gleason score, metastatic status at diagnosis, measurable disease, metastatic sites at cabazitaxel initiation, prior local and systemic treatments, docetaxel treatment setting and response, and post-cabazitaxel therapies.

Baseline laboratory values at cabazitaxel initiation included hemoglobin, neutrophil and lymphocyte counts, albumin, alkaline phosphatase (ALP), lactate dehydrogenase (LDH), and prostate-specific antigen (PSA). The neutrophil-to-lymphocyte ratio (NLR) was calculated as the absolute neutrophil count divided by the absolute lymphocyte count, and the prognostic nutritional index (PNI) was calculated as albumin (g/L) + 5 × absolute lymphocyte count (10^9^/L). Cabazitaxel-related variables included treatment line, number of cycles, dose modifications, best response, and adverse events. Availability of key baseline, outcome, and safety variables was recorded, with missingness summarized in Supplementary Table S1.

### 2.3. Cabazitaxel Treatment and Adverse Event Assessment

Cabazitaxel was administered intravenously at 20 or 25 mg/m^2^ every 3 weeks with oral prednisone, at the treating physician’s discretion. Granulocyte colony-stimulating factor (G-CSF) prophylaxis and other supportive treatments were used according to institutional practice. Treatment continued until radiologic or clinical progression, unacceptable toxicity, patient refusal, or physician decision. Treatment duration was individualized according to disease control, tolerability, patient preference, and clinical judgment. Specific patient-level reasons for cabazitaxel discontinuation were not captured as a standardized variable and could not be reliably reconstructed. Formal multidisciplinary tumor board approval was not routinely required for continuation beyond 10 cycles.

Hematologic adverse events were retrospectively assessed from medical records and laboratory results and graded according to the National Cancer Institute Common Terminology Criteria for Adverse Events, version 5.0. Available data for supportive care and treatment modification included primary G-CSF prophylaxis and cabazitaxel dose reduction. Safety outcomes were defined as cumulative patient-level events documented over the entire treatment course. Exact timing and cycle number of hematologic adverse events, treatment delays, patient time at risk, and dose intensity were not consistently available; therefore, exposure-adjusted incidence rates and time-to-first-event analyses could not be performed reliably. Nonhematologic adverse events were not systematically evaluated.

### 2.4. Study Endpoints and Response Assessment

The primary endpoint was OS from the predefined 8-month landmark to death from any cause. Secondary endpoints were conditional radiologic progression-free survival (rPFS), conditional PSA progression-free survival (PSA-PFS), objective response rate (ORR), disease control rate (DCR), PSA response, and safety.

Conditional rPFS was calculated from the 8-month landmark to radiologic progression or death, whichever occurred first, among patients who were alive and radiographically progression-free at the landmark. Conditional PSA-PFS was calculated from the 8-month landmark to PSA progression according to PCWG2 criteria among patients who were alive and had not experienced PSA progression by the landmark [17]. Patients who had already experienced the corresponding progression event before the landmark were excluded from the respective conditional analysis.

Imaging assessments were performed according to routine clinical practice rather than at predefined intervals. Radiologic response was retrospectively determined from radiology reports and clinical records using RECIST version 1.1 for measurable disease and PCWG2 criteria for bone lesions [17,19]. For rPFS, radiographic progression required documented imaging progression according to these criteria; clinical progression and treatment discontinuation were not classified as radiographic progression in the absence of documented imaging progression. Exact dates and intervals of serial imaging assessments were not consistently available; consequently, median imaging intervals could not be reliably calculated. ORR comprised complete or partial response among patients with measurable disease, whereas DCR comprised complete response, partial response, or stable disease. Best radiographic response was defined as the best response documented over the entire cabazitaxel treatment course. Because a common prespecified response assessment window and the exact timing of best response were unavailable, ORR and DCR were considered descriptive rather than comparative estimates of treatment efficacy. PSA response was defined as a ≥50% decline from baseline, confirmed at least 3 weeks later [17].

### 2.5. Statistical Analysis

Continuous variables were summarized as medians and interquartile ranges, and categorical variables as frequencies and percentages. The distribution of continuous variables was assessed using the Shapiro–Wilk test. Groups were compared using the Mann–Whitney U test for continuous variables and the chi-square test or Fisher’s exact test for categorical variables, as appropriate. 

Time-to-event outcomes were estimated using the Kaplan–Meier method and compared with the log-rank test. The primary OS analysis used the predefined 8-month landmark approach. Conditional rPFS and PSA-PFS were analyzed from the 8-month landmark among patients who remained free of the corresponding progression event at that time. These were considered conditional post-landmark outcomes rather than causal treatment effects.

Univariable and multivariable Cox proportional hazards models were used to evaluate associations between baseline prognostic factors and OS from the landmark. Variables with a *p*-value < 0.10 in univariable analysis or considered clinically relevant were considered for the multivariable model. Hazard ratios and 95% confidence intervals were reported. Given the sample size and number of events, a parsimonious model was constructed. Multivariable analyses were performed using complete cases for the variables included in each model. Clinically or biologically correlated variables were not entered simultaneously to reduce multicollinearity. The proportional hazards assumption was assessed using covariate-by-log(time since the landmark) interaction terms, with a statistically significant interaction considered evidence of non-proportional hazards. Multivariable models provided adjustment for available baseline prognostic factors but were not intended to control for time-dependent determinants of treatment continuation.

Treatment-era heterogeneity was explored by comparing the proportion receiving >10 cycles between 2011–2014 and 2015–2019 using Fisher’s exact test. An expanded sensitivity model additionally incorporated bone, lymph node, and visceral metastases and calendar year of cabazitaxel initiation as a continuous covariate. Missing-data sensitivity analyses compared landmark OS according to the availability of Gleason score and LDH and reassessed the unadjusted treatment duration estimate in the complete-case subset used for the parsimonious model; multiple imputation was not performed.

Sensitivity analyses included alternative 6- and 7-month landmarks in the full 98-patient source cohort and an exploratory 8-month analysis restricted to patients who were alive and radiographically progression-free at the landmark. OS was calculated from the corresponding landmark. 

Analyses were performed using IBM SPSS Statistics for Windows, version 23.0 (IBM Corp., Armonk, NY, USA). All tests were two-sided, and a *p*-value < 0.05 was considered statistically significant.

## 3. Results

### 3.1. Study Population and Baseline Characteristics

**Figure 1:**
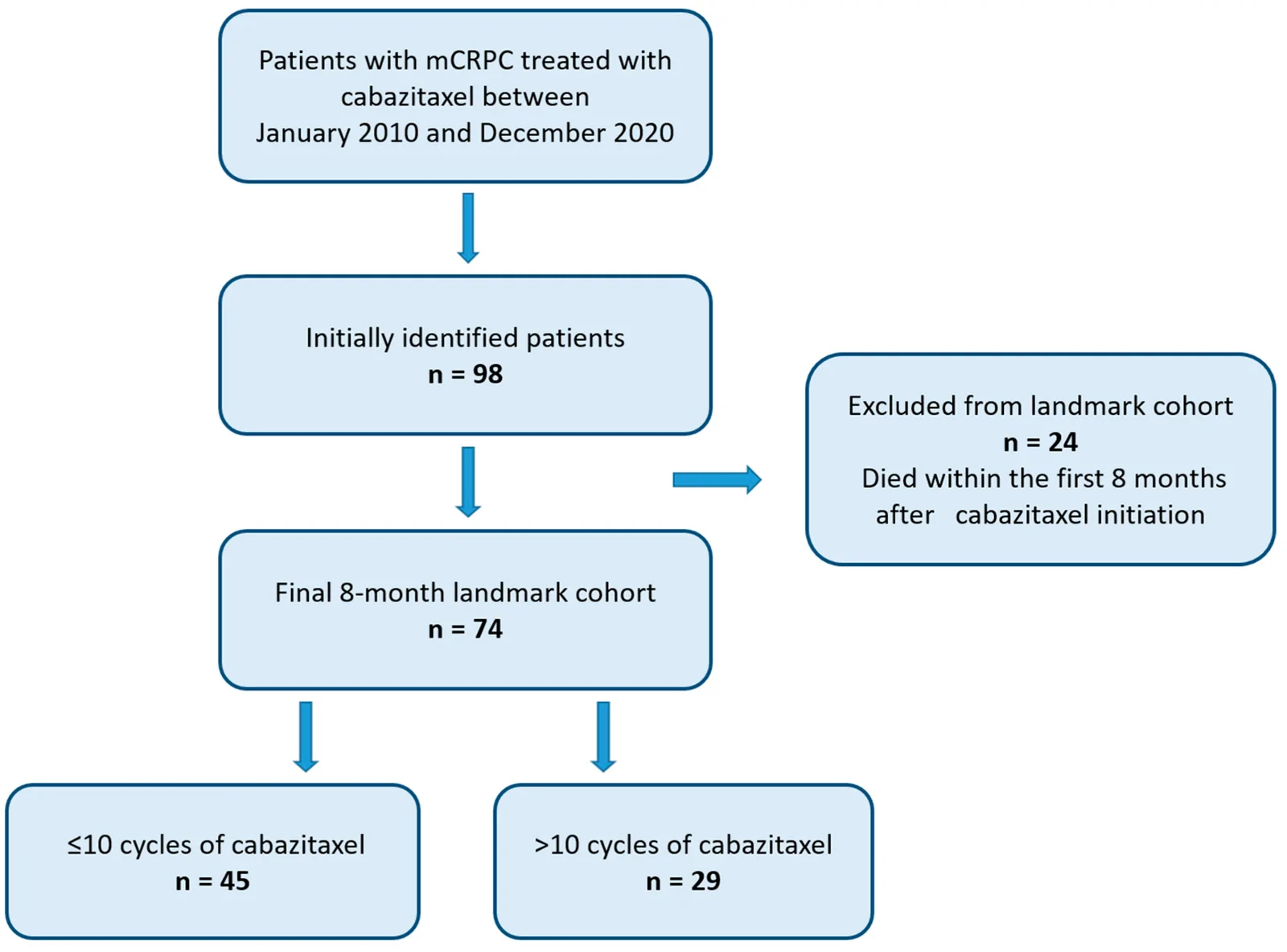
Flow diagram of patient selection and 8-month landmark cohort formation. A total of 98 patients with metastatic castration-resistant prostate cancer treated with cabazitaxel were initially identified. A total of 24 patients who died within the first 8 months after cabazitaxel initiation were excluded, leaving 74 patients in the final 8-month landmark cohort. Patients were categorized according to the total number of cabazitaxel cycles received as ≤10 cycles (n = 45) or >10 cycles (n = 29).

Of 98 initially identified patients, 74 were alive at the predefined 8-month landmark and constituted the final cohort; 45 (60.8%) ultimately received ≤10 cycles and 29 (39.2%) received >10 cycles of cabazitaxel (Figure 1). The median age at prostate cancer diagnosis was 61.0 years (IQR, 56.0–65.0), and 68.9% had metastatic disease at diagnosis. The Gleason score was available for 60 patients (81.1%), of whom 49 (81.7%) had a score ≥8; it could not be reliably retrieved for the remaining 14 patients. The patients had received a median of two prior systemic treatment lines (range, 1–4) and all had previously received docetaxel, predominantly in the mCRPC setting (87.8%), with a median of eight cycles (IQR, 6–12). Additional baseline and prior-treatment characteristics are summarized in Table 1.

### 3.2. Baseline Characteristics at Cabazitaxel Initiation and Treatment Exposure

Baseline demographic, clinical, and laboratory characteristics were generally comparable between groups (Table 2). Cabazitaxel was administered as second- or third-line treatment in 71.1% of patients in the ≤10-cycle group and 82.8% in the >10-cycle group (*p* = 0.254). Cabazitaxel in the treatment line following docetaxel was more frequent in the >10-cycle group (75.9% vs. 53.3%; *p* = 0.051), as was measurable disease (72.4% vs. 51.1%; *p* = 0.068). Lymph node metastases were significantly more common in the >10-cycle group (82.8% vs. 57.8%; *p* = 0.025), whereas bone metastases were numerically more frequent in the ≤10-cycle group (93.3% vs. 79.3%; *p* = 0.072); visceral metastasis rates were similar (26.7% vs. 17.2%; *p* = 0.347). Median cabazitaxel exposure was 6 cycles (range, 1–10) in the ≤10-cycle group and 15 cycles (range, 11–36) in the >10-cycle group.

### 3.3. Survival and Treatment Response Outcomes

At the data cutoff, all patients in the landmark cohort had died. For descriptive purposes, the median OS from cabazitaxel initiation was 20.4 months (95% CI, 16.7–24.1), median rPFS was 9.3 months (95% CI, 7.7–10.9), and median PSA-PFS was 6.9 months (95% CI, 4.9–8.9) in the landmark-selected cohort.

**Figure 2:**
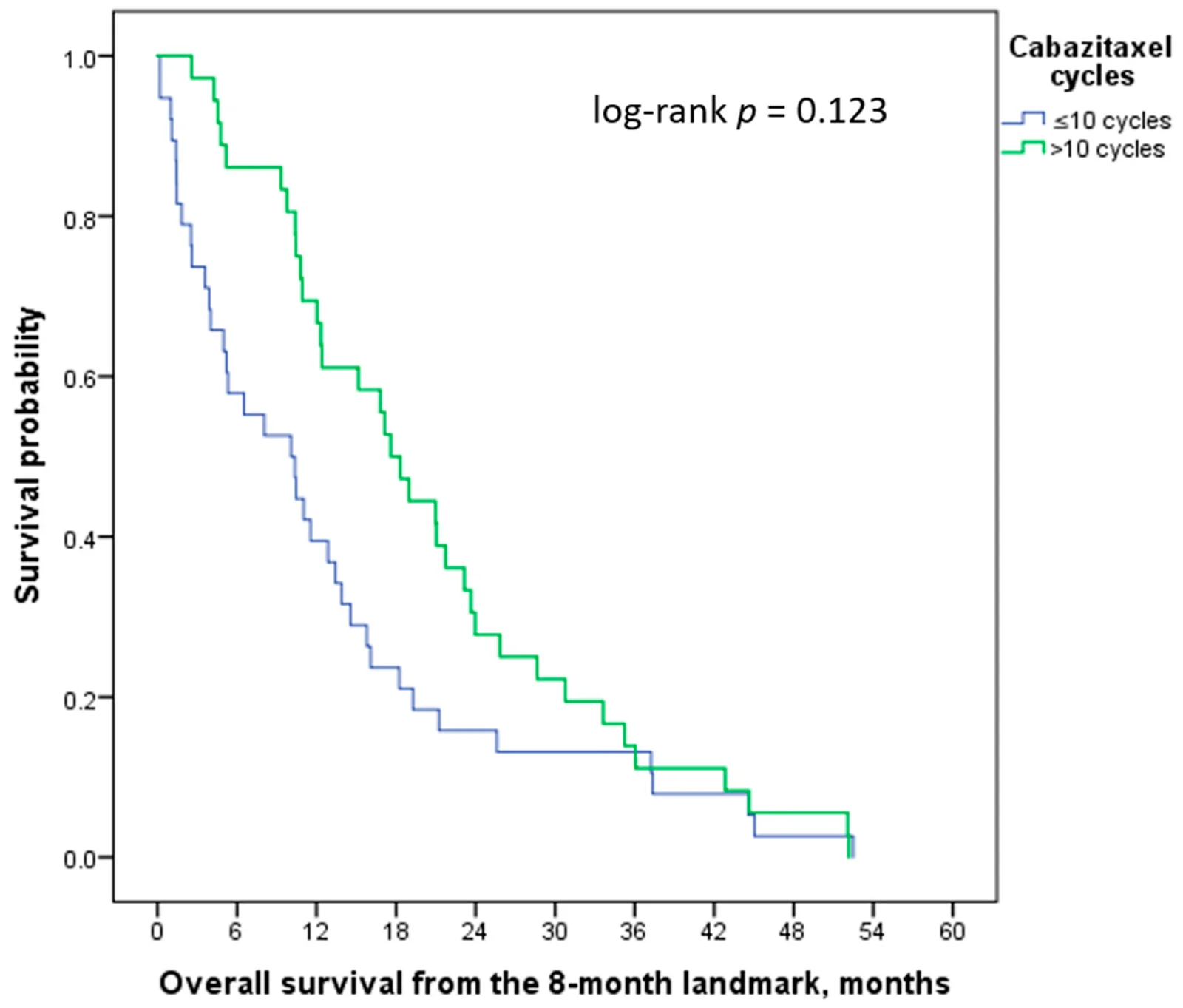
Kaplan–Meier estimates of overall survival from the predefined 8-month landmark according to cabazitaxel treatment duration. Median overall survival from the landmark was 10.4 months in the ≤10-cycle group and 17.6 months in the >10-cycle group (log-rank *p* = 0.123).

The median OS from cabazitaxel initiation was numerically longer in the >10-cycle group than in the ≤10-cycle group (25.6 months [95% CI, 22.9–28.3] vs. 18.4 months [95% CI, 12.4–24.3]; log-rank *p* = 0.123). In the primary landmark analysis, the median OS was 17.6 months (95% CI, 14.9–20.3) in the >10-cycle group and 10.4 months (95% CI, 4.4–16.3) in the ≤10-cycle group (log-rank *p* = 0.123) (Figure 2). 

**Figure 3:**
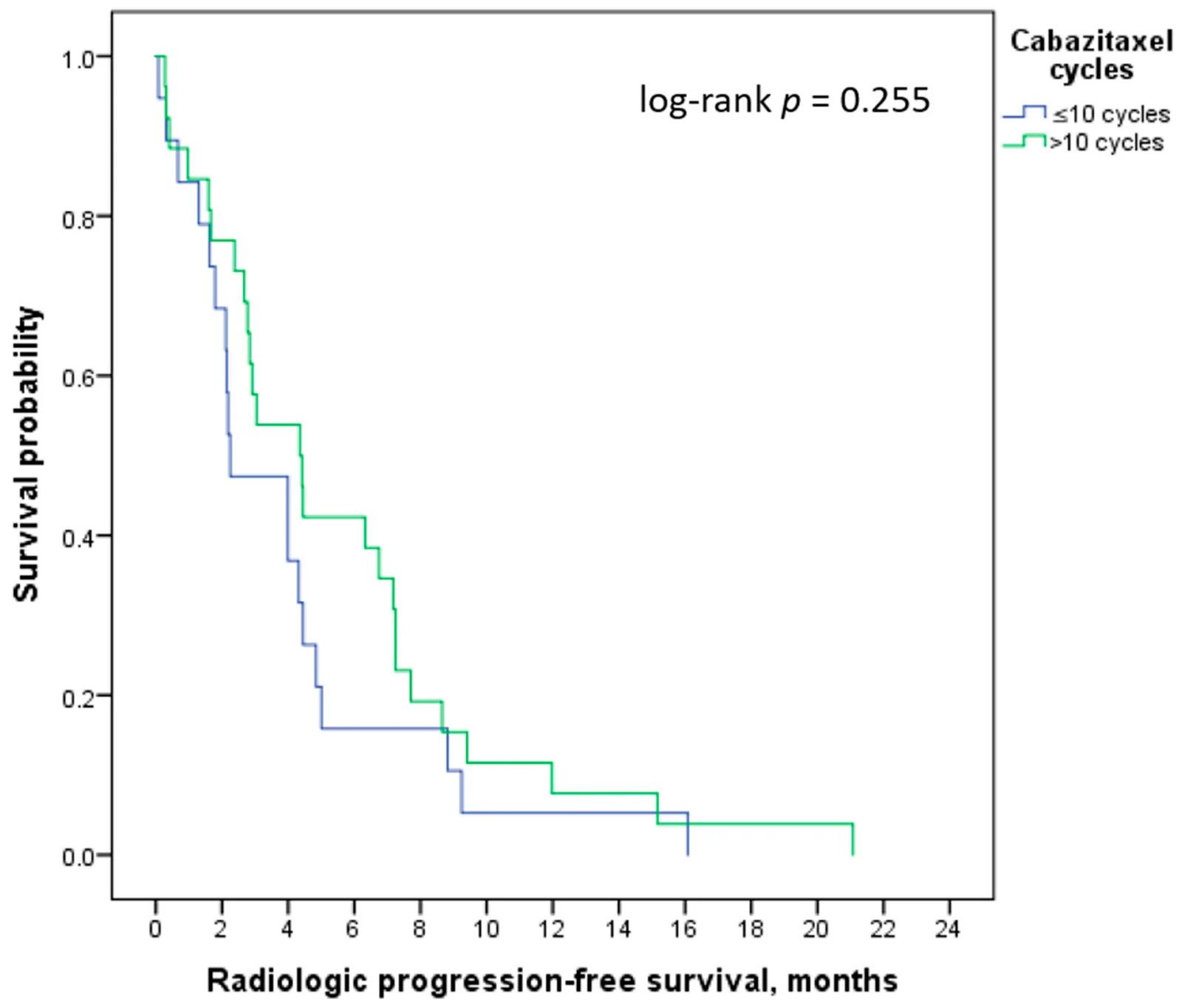
Kaplan–Meier estimates of conditional radiologic progression-free survival from the 8-month landmark according to cabazitaxel treatment duration. Median conditional rPFS was 2.3 months in the ≤10-cycle group and 4.4 months in the >10-cycle group (log-rank *p* = 0.255).

At the 8-month landmark, 45 patients were alive and radiographically progression-free (19 in the ≤10-cycle group and 26 in the >10-cycle group). Median conditional rPFS from the landmark was 2.3 months (95% CI, 0.3–4.2) in the ≤10-cycle group and 4.4 months (95% CI, 2.4–6.3) in the >10-cycle group (log-rank *p* = 0.255) (Figure 3). For conditional PSA-PFS, 32 patients had not experienced PSA progression by the landmark (15 in the ≤10-cycle group and 17 in the >10-cycle group). Median conditional PSA-PFS was 2.2 months (95% CI, 1.1–3.2) in the ≤10-cycle group and 4.2 months (95% CI, 1.8–6.6) in the >10-cycle group (log-rank *p* = 0.137).

Among patients with measurable disease, the ORR was 43.5% in the ≤10-cycle group and 52.4% in the >10-cycle group. The DCR based on best overall response was 66.7% (30/45) and 96.6% (28/29), respectively. Because response assessments were performed over unequal treatment durations and without a standardized common assessment window, the ORR and DCR were considered descriptive. Among patients with available PSA response data, ≥50% PSA declines were observed in 22 of 30 evaluable patients (73.3%) and 20 of 28 evaluable patients (71.4%), respectively. PSA response comparisons were also considered descriptive because evaluability differed substantially between groups (Table 3).

### 3.4. Sensitivity Analyses

Sensitivity analyses using alternative landmarks showed a consistent direction of association but varying effect estimates. At the 6-month landmark, 86 patients were included (57 ≤10 cycles and 29 >10 cycles), with an HR for death of 1.72 (95% CI, 1.10–2.71; *p* = 0.017) for ≤10 versus >10 cycles. At the 7-month landmark, 82 patients were included (53 and 29, respectively), with an HR of 1.64 (95% CI, 1.04–2.59; *p* = 0.033). At the predefined 8-month landmark, 74 patients were included (45 and 29, respectively), with an HR of 1.45 (95% CI, 0.90–2.32; *p* = 0.125).

Cabazitaxel initiation in the full 98-patient source cohort occurred between 2011 and 2019. In total, 17 of 51 patients (33.3%) initiating cabazitaxel during 2011–2014 and 12 of 47 patients (25.5%) during 2015–2019 ultimately received >10 cycles, with no statistically significant difference between calendar periods (*p* = 0.507).

Pre-landmark radiographic progression differed markedly between treatment duration groups, occurring in 26 of 45 patients (57.8%) in the ≤10-cycle group and 3 of 29 patients (10.3%) in the >10-cycle group (*p* < 0.001). When the analysis was restricted to patients who were both alive and radiographically progression-free at the 8-month landmark (n = 45; 19 in the ≤10-cycle group and 26 in the >10-cycle group), the HR for subsequent OS was 0.92 for ≤10 versus >10 cycles (95% CI, 0.50–1.68; *p* = 0.785).

### 3.5. Univariable and Multivariable Cox Regression Analyses for Overall Survival

In univariable analysis, docetaxel use in the mHSPC setting was associated with a higher hazard of death than use in the mCRPC setting (HR, 2.58; 95% CI, 1.25–5.34; *p* = 0.011). Higher hemoglobin (HR, 0.81 per 1 g/dL increase; 95% CI, 0.69–0.97; *p* = 0.019) and albumin (HR, 0.49 per 1 g/dL increase; 95% CI, 0.26–0.89; *p* = 0.019) were associated with a lower hazard of death, whereas elevated ALP was associated with a higher hazard (HR, 1.96; 95% CI, 1.18–3.26; *p* = 0.010). The ≤10-cycle versus >10-cycle classification was associated with a numerically higher hazard of death (HR, 1.45; 95% CI, 0.90–2.32; *p* = 0.125).

The parsimonious multivariable model included cabazitaxel cycle group, hemoglobin, and elevated ALP and was based on 69 patients with complete data. Albumin and docetaxel treatment setting were not retained because of overlapping prognostic information and the limited mHSPC subgroup size, respectively. In this complete-case subset, the unadjusted treatment duration estimate (HR for ≤10 vs. >10 cycles, 1.41; 95% CI, 0.87–2.29; *p* = 0.167) was similar to that in the full 74-patient landmark cohort (HR, 1.45; 95% CI, 0.90–2.32; *p* = 0.125). After baseline prognostic adjustment, the HR for ≤10 versus >10 cycles was 1.82 (95% CI, 1.08–3.06; *p* = 0.024). Elevated ALP was associated with a higher hazard of death (HR, 2.25; 95% CI, 1.30–3.91; *p* = 0.004), whereas higher hemoglobin was associated with a lower hazard (HR, 0.82 per 1 g/dL increase; 95% CI, 0.69–0.97; *p* = 0.022) (Table 4).

In the expanded sensitivity model incorporating bone, lymph node, and visceral metastases and calendar year of cabazitaxel initiation, the treatment duration estimate was attenuated and was no longer statistically significant (HR for ≤10 vs. >10 cycles, 1.58; 95% CI, 0.91–2.74; *p* = 0.105). In missing-data sensitivity analyses, availability of Gleason score and LDH was not associated with landmark OS (HR for missing vs. available Gleason score, 0.98; 95% CI, 0.55–1.76; *p* = 0.946; HR for missing vs. available LDH, 1.01; 95% CI, 0.64–1.60; *p* = 0.972). 

Assessment of the proportional hazards assumption using covariate-by-log(time) interactions indicated non-proportional hazards for treatment duration (interaction *p* = 0.024) but not for hemoglobin (*p* = 0.138) or elevated ALP (*p* = 0.762). Accordingly, the treatment duration HR should be interpreted as a model-based summary association over follow-up rather than as a constant relative hazard.

### 3.6. Safety and Post-Cabazitaxel Treatments

Primary G-CSF prophylaxis was used at similar rates in the ≤10-cycle and >10-cycle groups (95.1% vs. 92.9%; *p* = 0.693). Among patients with available safety data, the crude cumulative proportions experiencing grade ≥3 neutropenia (14.0% vs. 48.3%; *p* = 0.001) and febrile neutropenia (4.7% vs. 27.6%; *p* = 0.006) were higher in the >10-cycle group, whereas dose reduction rates were similar (45.2% vs. 50.0%; *p* = 0.696) (Table 5). These comparisons were not adjusted for the substantially longer treatment exposure in the >10-cycle group.

After cabazitaxel discontinuation, platinum–etoposide was the most common subsequent treatment in the ≤10-cycle group (33.3%), whereas abiraterone acetate was most common in the >10-cycle group (41.4%). No further systemic treatment was administered to 17.8% and 24.1% of patients, respectively (Table 6).

## 4. Discussion

In this retrospective study, we evaluated survival outcomes and safety according to cabazitaxel treatment duration in patients with mCRPC using a predefined 8-month landmark approach intended to reduce early immortal time bias. Patients who ultimately received >10 cycles had numerically longer landmark OS than those who received ≤10 cycles, with median estimates of 17.6 months (95% CI, 14.9–20.3) and 10.4 months (95% CI, 4.4–16.3), respectively. The unadjusted treatment duration HR for ≤10 versus >10 cycles was 1.45 (95% CI, 0.90–2.32). The apparent survival difference was not consistently maintained across sensitivity analyses and should be interpreted cautiously in view of treatment selection and responder enrichment biases. Higher crude cumulative proportions of grade ≥3 neutropenia and febrile neutropenia were observed in the >10-cycle group. To our knowledge, this is the first study to evaluate cabazitaxel treatment beyond 10 cycles using a predefined landmark approach. Overall, cabazitaxel treatment beyond 10 cycles appears feasible in selected patients, but the observed outcomes are more appropriately interpreted as reflecting a favorable treatment trajectory than as evidence that continuation beyond 10 cycles improves survival.

Our findings are broadly consistent with previous retrospective studies evaluating cabazitaxel treatment beyond 10 cycles. When OS was calculated from cabazitaxel initiation, median OS was numerically longer among patients who received >10 cycles in our cohort (25.6 vs. 18.4 months). Noronha et al. reported sustained clinical benefit with manageable toxicity beyond 10 cycles [15], whereas Shiota et al. observed longer progression-free and overall survival [14], and Al-Mansouri et al. reported longer median OS with >10 versus 4–10 cycles (14 vs. 7 months; HR, 0.28; *p* = 0.01) [13]. A post hoc analysis of compassionate use and expanded access programs and the CAPRISTANA registry also found that 13.0% of patients received ≥11 cycles [20]. Collectively, these data support the feasibility of cabazitaxel treatment beyond 10 cycles in selected patients.

Interpreting our results requires consideration of both the landmark-selected study population and the future-derived definition of treatment duration. Previous studies were based mainly on small retrospective cohorts [13,14,15] and were therefore susceptible to treatment selection bias and immortal time bias [16]. The predefined 8-month landmark reduced early immortal time bias by restricting the analyses to patients alive at a common time point [16,18]. Sensitivity analyses using 6-, 7-, and 8-month landmarks showed a consistent direction of association with attenuation at later landmarks. Across the complementary Cox models, the HRs for ≤10 versus >10 cycles were 1.45 (95% CI, 0.90–2.32) in the unadjusted landmark analysis, 1.82 (95% CI, 1.08–3.06) in the parsimonious baseline-adjusted model, and 1.58 (95% CI, 0.91–2.74) in the expanded sensitivity model, indicating model-dependent estimates with substantial uncertainty. Because the proportional hazards assumption was not satisfied for treatment duration, these HRs should be interpreted as model-based summary associations over follow-up rather than as constant relative hazards. However, classification according to the total number of cycles ultimately received did not eliminate time-dependent treatment selection. The >10-cycle group was enriched for patients who remained alive, maintained disease control, tolerated treatment, and were able to continue therapy, whereas patients with early progression were more likely to discontinue earlier. This was reflected by the marked imbalance in pre-landmark radiographic progression, and the subsequent OS difference was no longer evident when the analysis was restricted to patients who were alive and radiographically progression-free at the 8-month landmark. Accordingly, prolonged cabazitaxel exposure should be interpreted primarily as a marker of a favorable treatment trajectory rather than evidence that continuation itself improves survival. 

The clinical question addressed by this study should be distinguished from the causal question of whether cabazitaxel should be continued beyond 10 cycles. Ideally, this question would be evaluated in a target trial framework comparing continuation versus discontinuation among patients who are alive, progression-free, clinically fit, and eligible for either strategy at a prespecified decision point near cycle 10 [21]. Our retrospective dataset could not reliably emulate such a trial because treatment duration was defined by the total number of cycles ultimately received and contemporaneous data on cycle timing, performance status, treatment interruptions, and clinical eligibility were not consistently available. Accordingly, our findings should be interpreted as describing the prognosis associated with different treatment trajectories rather than the causal effect of treatment continuation.

Differences in metastatic distribution may also have influenced outcomes. Lymph node metastases were more frequent in the >10-cycle group, whereas bone metastases were numerically more common in the ≤10-cycle group. Lymph node-only disease is generally associated with a more favorable prognosis than bone or visceral involvement, whereas bone metastases, elevated ALP, and lower hemoglobin are adverse prognostic features in mCRPC [22,23]. However, nodal involvement in our cohort did not necessarily represent lymph node-only disease. Although metastatic sites were not included in the parsimonious multivariable model, bone, lymph-node, and visceral metastases were incorporated into the expanded sensitivity model. Residual confounding related to metastatic distribution nevertheless cannot be excluded. Moreover, such baseline adjustment does not capture time-dependent determinants of treatment continuation, including early treatment sensitivity, tolerability, performance status, and physician selection.

Conditional analyses from the 8-month landmark yielded median rPFS estimates of 2.3 months (95% CI, 0.3–4.2) in the ≤10-cycle group and 4.4 months (95% CI, 2.4–6.3) in the >10-cycle group; corresponding median PSA-PFS estimates were 2.2 months (95% CI, 1.1–3.2) and 4.2 months (95% CI, 1.8–6.6), respectively. These findings are consistent with responder enrichment and reverse causation, as patients with sustained disease control were more likely to remain on cabazitaxel long enough to receive >10 cycles. Accordingly, these progression outcomes should not be interpreted as evidence that treatment beyond 10 cycles improved disease control. Similar caution applies to the ORR and DCR, which were based on best response over unequal treatment durations without a standardized assessment window. Patients receiving more cycles therefore had greater opportunity to demonstrate response or stable disease, and these outcomes should be interpreted descriptively rather than as evidence of greater treatment efficacy.

Hematologic adverse events remain an important consideration. The crude cumulative proportions of grade ≥3 neutropenia and febrile neutropenia were higher in the >10-cycle group, whereas dose reduction and primary G-CSF prophylaxis rates were similar between groups. However, these comparisons represent cumulative proportions over markedly different treatment exposures and should not be interpreted as exposure-adjusted toxicity risks. The higher cumulative event proportions with >10 cycles may partly reflect greater treatment exposure, whereas patients who developed clinically important toxicity and discontinued early could not subsequently enter the >10-cycle group, introducing tolerability-based selection. Previous studies likewise support the feasibility of cabazitaxel treatment beyond 10 cycles with careful monitoring and individualized toxicity management [13,14,15,20]. When cabazitaxel treatment extends beyond 10 cycles, ongoing assessment of both clinical benefit and hematologic toxicity remains important.

Post-cabazitaxel treatment patterns may also provide indirect information regarding underlying disease biology. Platinum–etoposide was more frequently administered in the ≤10-cycle group, which may have reflected clinical concern for aggressive-variant prostate cancer or neuroendocrine differentiation [24]. Such phenotypes may be associated with earlier treatment resistance, shorter cabazitaxel exposure, and poorer survival. Because metastatic biopsy findings and neuroendocrine markers were not consistently available, platinum–etoposide use may have served as a surrogate for unmeasured aggressive disease biology rather than simply reflecting a difference in subsequent treatment choice. In addition, discontinuation because of progression, toxicity, patient decision, or physician-directed treatment change may represent distinct clinical trajectories, adding heterogeneity to the ≤10-cycle group and further limiting attribution of outcome differences to treatment duration.

The study period encompassed substantial changes in mCRPC management. Although the proportion of patients receiving >10 cycles did not differ between the examined calendar periods and calendar year was included as a continuous covariate in the expanded sensitivity model, residual treatment-era confounding cannot be excluded. The cohort also largely preceded widespread availability of PARP inhibitors, PSMA-targeted radioligand therapy, and other biomarker-directed approaches [3,4,5], and limited treatment alternatives may have contributed to prolonged cabazitaxel exposure in some patients. Nevertheless, access to newer therapies remains heterogeneous, and cabazitaxel continues to be an important option for patients previously treated with docetaxel and an ARPI [2,4,5,6].

Several limitations should be acknowledged. The retrospective single-center design and modest sample size increase the potential for selection bias and residual confounding. Although the landmark approach reduced early immortal time bias, treatment groups remained defined by the total number of cycles ultimately received, leaving time-dependent treatment selection and responder enrichment. Exact cycle timing, treatment interruptions, early on-treatment variables, and reasons for discontinuation were not consistently available, precluding reliable evaluation of the clinical decision to continue treatment beyond 10 cycles. Unmeasured aggressive-variant or neuroendocrine disease biology may also have influenced treatment duration and outcomes. Missing data, particularly for LDH and PSA response evaluability, may have introduced additional bias; multiple imputation was not performed because of the small sample size and uncertain missing-data mechanism. Conditional progression analyses involved smaller event-free subsets, and nonstandardized imaging may have introduced assessment time bias. In addition, incomplete cycle-specific safety and exposure data precluded exposure-adjusted toxicity analyses, and nonhematologic toxicities were not systematically assessed. Finally, the lack of external validation limits the generalizability of our findings, which should therefore be considered hypothesis-generating.

## 5. Conclusions

In this retrospective 8-month landmark analysis, patients who ultimately received >10 cycles of cabazitaxel had a numerically longer median OS than those who received ≤10 cycles, although the primary landmark comparison was not statistically significant. Cabazitaxel treatment beyond 10 cycles appeared feasible in selected patients. However, future-derived exposure classification based on the total number of cycles ultimately received and responder enrichment limit causal interpretation. Accordingly, the observed outcomes are more appropriately interpreted as reflecting a favorable treatment trajectory rather than evidence of a survival benefit from continuation beyond 10 cycles. Higher crude cumulative proportions of grade ≥3 neutropenia and febrile neutropenia were observed in the >10-cycle group. These findings should therefore be considered hypothesis-generating. Prospective randomized studies comparing continuation versus discontinuation among patients eligible to continue treatment at a prespecified decision point are needed to determine the clinical value of treatment beyond 10 cycles.

## Figures and Tables

**Table 1:** Baseline characteristics and prior treatment history of the 8-month landmark cohort.

Characteristic	Overall Cohort (n = 74)
Age at prostate cancer diagnosis, years, median (IQR)	61.0 (56.0–65.0)
Age group	
<65 years	50 (67.6%)
≥65 years	24 (32.4%)
Gleason score (n = 60)	
<8	11 (18.3%)
≥8	49 (81.7%)
Prostate surgery	11 (14.9%)
Definitive or salvage radiotherapy	17 (23.0%)
Orchiectomy	19 (25.7%)
Metastatic disease at diagnosis	51 (68.9%)
Number of prior systemic treatment lines, median (range)	2 (1–4)
Prior systemic treatment sequence	
First-line treatment	
ADT alone	52 (70.3%)
Docetaxel	21 (28.4%)
Abiraterone acetate	1 (1.4%)
Second-line treatment (n = 67)	
Docetaxel	53 (79.1%)
Abiraterone acetate	11 (16.4%)
Enzalutamide	2 (3.0%)
Mitoxantrone	1 (1.5%)
Third-line treatment (n = 18)	
Abiraterone acetate	9 (50.0%)
Lutetium-based therapy	4 (22.2%)
Mitoxantrone	4 (22.2%)
Enzalutamide	1 (5.6%)
Prior docetaxel treatment setting	
mHSPC	9 (12.2%)
mCRPC	65 (87.8%)
Prior docetaxel cycles, median (IQR)	8 (6–12)
Best radiologic response to prior docetaxel	
Partial response	34 (46.0%)
Stable disease	30 (40.5%)
Progressive disease	10 (13.5%)

Abbreviations: ADT, androgen deprivation therapy; IQR, interquartile range; mCRPC, metastatic castration-resistant prostate cancer; mHSPC, metastatic hormone-sensitive prostate cancer. Data are presented as n (%) unless otherwise indicated. One patient received abiraterone acetate as fourth-line systemic therapy before cabazitaxel initiation.

**Table 2:** Baseline characteristics at cabazitaxel initiation in the 8-month landmark cohort according to cabazitaxel treatment duration.

Characteristic	≤10 Cycles (n = 45)	>10 Cycles (n = 29)	*p*-Value
Age at cabazitaxel initiation, years, median (IQR)	66.0 (58.0–74.0)	67.0 (63.0–71.0)	0.864
Age group at cabazitaxel initiation			0.434
<70 years	27 (60.0%)	20 (69.0%)	
≥70 years	18 (40.0%)	9 (31.0%)	
Gleason score (n = 60)			0.683
<8	6 (16.7%)	5 (20.8%)	
≥8	30 (83.3%)	19 (79.2%)	
PSA level, ng/mL, median (IQR)	91.8 (43.7–217.0)	85.8 (44.5–152.4)	0.718
Metastatic disease at diagnosis	33 (73.3%)	18 (62.1%)	0.307
Cabazitaxel treatment following docetaxel	24 (53.3%)	22 (75.9%)	0.051
Cabazitaxel treatment line			0.254
Second or third line	32 (71.1%)	24 (82.8%)	
Fourth or fifth line	13 (28.9%)	5 (17.2%)	
Hemoglobin level, g/dL, median (IQR)	12.1 (11.2–13.0)	12.3 (11.0–13.1)	0.845
NLR, median (IQR)	2.6 (2.0–3.1)	2.2 (1.8–3.3)	0.243
PNI, median (IQR)	50.0 (47.0–54.2)	51.9 (49.0–55.7)	0.359
Albumin level, g/dL, median (IQR)	4.1 (3.8–4.3)	4.2 (3.9–4.3)	0.213
Elevated ALP	25 (61.0%)	16 (57.1%)	0.750
LDH level, U/L, median (IQR) (n = 40)	304 (227–452)	312 (199–370)	0.374
Measurable disease	23 (51.1%)	21 (72.4%)	0.068
Bone metastases	42 (93.3%)	23 (79.3%)	0.072
Lymph node metastases	26 (57.8%)	24 (82.8%)	0.025
Visceral metastases	12 (26.7%)	5 (17.2%)	0.347

Abbreviations: ALP, alkaline phosphatase; IQR, interquartile range; LDH, lactate dehydrogenase; NLR, neutrophil-to-lymphocyte ratio; PNI, prognostic nutritional index; PSA, prostate-specific antigen. Data are presented as n (%) unless otherwise indicated. Percentages for variables with missing data were calculated using available cases. Elevated ALP was defined as a value above the institutional upper limit of normal. *p*-values were calculated using the Mann–Whitney U test for continuous variables and the chi-square test or Fisher’s exact test for categorical variables, as appropriate.

**Table 3:** Survival and treatment response outcomes in the 8-month landmark cohort according to cabazitaxel treatment duration.

Outcome	≤10 Cycles (n = 45)	>10 Cycles (n = 29)	*p*-Value
Best radiologic response			—
Partial response	10 (22.2%)	11 (37.9%)	
Stable disease	20 (44.4%)	17 (58.6%)	
Progressive disease	15 (33.3%)	1 (3.4%)	
Objective response rate *	10 (43.5%)	11 (52.4%)	—
Disease control rate	30 (66.7%)	28 (96.6%)	—
PSA response, n (%)			—
≥50% decline	22 (73.3%)	20 (71.4%)	
<50% decline	8 (26.7%)	8 (28.6%)	
Conditional rPFS, months (95% CI)	2.3 (0.3–4.2)	4.4 (2.4–6.3)	0.255
Conditional PSA-PFS, months (95% CI)	2.2 (1.1–3.2)	4.2 (1.8–6.6)	0.137
Median OS from cabazitaxel initiation, months (95% CI)	18.4 (12.4–24.3)	25.6 (22.9–28.3)	0.123
Median OS from the 8-month landmark, months (95% CI)	10.4 (4.4–16.3)	17.6 (14.9–20.3)	0.123

Abbreviations: CI, confidence interval; OS, overall survival; PSA, prostate-specific antigen; PSA-PFS, prostate-specific antigen progression-free survival; rPFS, radiologic progression-free survival. Data are presented as n (%) unless otherwise indicated. * Objective response rate was calculated among patients with measurable disease at baseline (≤10 cycles: n = 23; >10 cycles: n = 21). ORR and DCR reflect best response over the treatment course and are presented descriptively because treatment exposure and response assessment opportunities differed between groups. PSA response was evaluable in 30 and 28 patients, respectively, and is presented descriptively because evaluability differed between groups. Conditional rPFS included patients alive and radiographically progression-free at the 8-month landmark (n = 19 and 26), whereas conditional PSA-PFS included patients alive without prior PSA progression (n = 15 and 17); both were calculated from the landmark and compared using the log-rank test.

**Table 4:** Univariable and multivariable Cox regression analyses for overall survival from the 8-month landmark.

Variable	Univariable Analysis		Multivariable Analysis	
	HR (95% CI)	*p*-Value	HR (95% CI)	*p*-Value
Docetaxel treatment setting (mCRPC vs. mHSPC)	2.58 (1.25–5.34)	0.011	—	—
Hemoglobin level (g/dL)	0.81 (0.69–0.97)	0.019	0.82 (0.69–0.97)	0.022
Albumin (g/dL)	0.49 (0.26–0.89)	0.019	—	—
Elevated ALP (no vs. yes)	1.96 (1.18–3.26)	0.010	2.25 (1.30–3.91)	0.004
Cabazitaxel cycles (>10 vs. ≤10)	1.45 (0.90–2.32)	0.125	1.82 (1.08–3.06)	0.024
Bone metastasis (no vs. yes)	1.85 (0.88–3.88)	0.104	—	—

Abbreviations: ALP, alkaline phosphatase; CI, confidence interval; HR, hazard ratio; mCRPC, metastatic castration-resistant prostate cancer; mHSPC, metastatic hormone-sensitive prostate cancer. For categorical variables, the first category listed in parentheses was used as the reference category; HRs represent the hazard for the second category relative to the first. For continuous variables, HRs are presented per one-unit increase. Variables not included in the multivariable model are indicated by an em dash. The multivariable analysis included 69 patients with complete data for the variables entered into the model; all 69 patients experienced the event.

**Table 5:** Hematologic adverse events and dose modifications in the 8-month landmark cohort according to cabazitaxel treatment duration.

Safety Outcome	≤10 Cycles (n = 45)	>10 Cycles (n = 29)	*p*-Value
Primary G-CSF prophylaxis	39/41 (95.1%)	26/28 (92.9%)	0.693
Dose reduction	19 (45.2%)	14 (50.0%)	0.696
Grade ≥3 neutropenia	6 (14.0%)	14 (48.3%)	0.001
Febrile neutropenia	2 (4.7%)	8 (27.6%)	0.006

Abbreviation: G-CSF, granulocyte colony-stimulating factor. Data are presented as n (%) unless otherwise indicated. Percentages were calculated among patients with available data: primary G-CSF prophylaxis, n = 41 and n = 28; dose reduction, n = 42 and n = 28; grade ≥3 neutropenia and febrile neutropenia, n = 43 and n = 29 in the ≤10-cycle and >10-cycle groups, respectively. Hematologic adverse event values represent crude cumulative patient-level proportions over the entire treatment course and are not exposure-adjusted incidence rates.

**Table 6:** First subsequent systemic treatment after cabazitaxel discontinuation in the 8-month landmark cohort according to cabazitaxel treatment duration.

Subsequent Systemic Treatment	≤10 Cycles (n = 45)	>10 Cycles (n = 29)
Platinum–etoposide chemotherapy	15 (33.3%)	4 (13.8%)
Abiraterone acetate	9 (20.0%)	12 (41.4%)
Lutetium-based therapy	6 (13.3%)	3 (10.3%)
Enzalutamide	1 (2.2%)	3 (10.3%)
Mitoxantrone	3 (6.7%)	0 (0.0%)
Other therapies *	3 (6.7%)	0 (0.0%)
No further treatment	8 (17.8%)	7 (24.1%)

Data are presented as n (%). Each patient was classified according to the first systemic treatment received after cabazitaxel discontinuation. * Other therapies included docetaxel rechallenge, pembrolizumab, and vinorelbine.

## Data Availability

The data supporting the findings of this study are available from the corresponding author upon reasonable request. The data are not publicly available due to patient privacy and ethical restrictions.

## Supplementary Materials

The following supporting information can be downloaded at: https://www.mdpi.com/article/10.3390/jcm15186966/s1, Table S1. Availability and missingness of key baseline, outcome, and safety variables according to cabazitaxel treatment duration.

## Abbreviations

The following abbreviations are used in this manuscript:

ADT, androgen deprivation therapy; ALP, alkaline phosphatase; ARPI, androgen receptor pathway inhibitor; CI, confidence interval; CR, complete response; CTCAE, Common Terminology Criteria for Adverse Events; DCR, disease control rate; G-CSF, granulocyte colony-stimulating factor; HR, hazard ratio; IQR, interquartile range; LDH, lactate dehydrogenase; mCRPC, metastatic castration-resistant prostate cancer; mHSPC, metastatic hormone-sensitive prostate cancer; NLR, neutrophil-to-lymphocyte ratio; ORR, objective response rate; OS, overall survival; PARP, poly(ADP-ribose) polymerase; PCWG2, Prostate Cancer Working Group 2; PFS, progression-free survival; PNI, prognostic nutritional index; PR, partial response; PSA, prostate-specific antigen; PSA-PFS, prostate-specific antigen progression-free survival; PSMA, prostate-specific membrane antigen; RECIST, Response Evaluation Criteria in Solid Tumors; rPFS, radiologic progression-free survival; SD, stable disease.
