# Supplementary material for: Association of Cabazitaxel Treatment Beyond 10 Cycles with Survival Outcomes in Metastatic Castration-Resistant Prostate Cancer: A Retrospective 8-Month Landmark Analysis
Source: J Clin Med. 2026 Sep 8;15(18):6966. doi: 10.3390/jcm15186966 (PMC13607230; doi:10.3390/jcm15186966)
Supplement: Supplementary file 1 [file jcm-15-06966-s001.zip › Supplementary Table S1.pdf]

Supplementary Table S1. Availability and Missingness of Key Baseline, Outcome, and Safety Variables According to Cabazitaxel Treatment Duration

**Variable**	**Available overall**	**Missing ≤10 cycles**	**Missing >10 cycles**
Gleason score	60/74 (81.1%)	9/45 (20.0%)	5/29 (17.2%)
Baseline PSA	73/74 (98.6%)	1/45 (2.2%)	0/29 (0.0%)
Hemoglobin level	69/74 (93.2%)	4/45 (8.9%)	1/29 (3.4%)
Albumin level	69/74 (93.2%)	4/45 (8.9%)	1/29 (3.4%)
Alkaline phospatase	69/74 (93.2%)	4/45 (8.9%)	1/29 (3.4%)
LDH	40/74 (54.1%)	22/45 (48.9%)	12/29 (41.4%)
PSA response	58/74 (78.4%)	15/45 (33.3%)	1/29 (3.4%)
Primary G-CSF prophylaxis	69/74 (93.2%)	4/45 (8.9%)	1/29 (3.4%)
Dose reduction	70/74 (94.6%)	3/45 (6.7%)	1/29 (3.4%)
Grade ≥3 neutropenia	72/74 (97.3%)	2/45 (4.4%)	0/29 (0.0%)
Febrile neutropenia	72/74 (97.3%)	2/45 (4.4%)	0/29 (0.0%)

Abbreviations: G-CSF, granulocyte colony-stimulating factor; LDH, lactate dehydrogenase; PSA, prostate-specific antigen. Data are presented as n/N (%). Percentages in the "Available overall" column were calculated using the total landmark cohort (N = 74), whereas percentages for missing data were calculated within each treatment-duration group.
